# Composition of the metabolomic bio-coronas isolated from *Ocimum sanctum* and *Rubia tinctorum*

**DOI:** 10.1186/s13104-020-05420-8

**Published:** 2021-01-06

**Authors:** Jasmina Kurepa, Jan A. Smalle

**Affiliations:** Plant Physiology, Biochemistry, Molecular Biology Program, Department of Plant and Soil Sciences, College of Agriculture, Food and Environment, University of Kentucky, Lexington, KY 40546 USA

**Keywords:** Titanium dioxide nanoparticles, Flavonoids, *Ocimum sanctum*, Tulsi, Holy basil, *Rubia tinctorum*, Common madder, Lipids

## Abstract

**Objective:**

Nanoharvesting from intact plants, organs, and cultured cells is a method in which nanoparticles are co-incubated with the target tissue, which leads to the internalization of nanoparticles. Internalized nanoparticles are coated in situ with specific metabolites that form a dynamic surface layer called a bio-corona. Our previous study showed that metabolites that form the bio-corona around anatase TiO_2_ nanoparticles incubated with leaves of the model plant *Arabidopsis thaliana* are enriched for flavonoids and lipids. The present study focused on the identification of metabolites isolated by nanoharvesting from two medicinal plants, *Ocimum sanctum* (Tulsi) and *Rubia tinctorum* (common madder).

**Results:**

To identify metabolites that form the bio-corona, Tulsi leaves and madder roots were incubated with ultra-small anatase TiO_2_ nanoparticles, the coated nanoparticles were collected, and the adsorbed molecules were released from the nanoparticle surface and analyzed using an untargeted metabolomics approach. Similar to the results in which Arabidopsis tissue was used as a source of metabolites, TiO_2_ nanoparticle bio-coronas from Tulsi and madder were enriched for flavonoids and lipids, suggesting that nanoharvesting has a wide-range application potential. The third group of metabolites enriched in bio-coronas isolated from both plants were small peptides with C-terminal arginine and lysine residues.

## Introduction

The atoms at the surface of nanoparticles (NPs) have an asymmetrical environment, and as a result, an asymmetrical force field that gives rise to unstable surface energy [1]. Adsorption of molecules from the immediate surrounding of the NPs and formation of the corona is a natural process that reduces this surface energy and leads to stabilization of the NPs [1]. The excess surface energy of small (≤ 20 nm) anatase TiO_2_ NPs is best dissipated by adsorption of enediols (e.g., catechols) and other ortho-substituted bidentate compounds [2–4]. Plants are a rich source of compounds that can function as enediol ligands, many of which are valuable nutraceuticals and therapeuticals with structures that are too complex for chemical synthesis [5]. We have previously shown that when plant tissue is co-incubated with small anatase TiO_2_ NPs, NPs are taken up by plant cells, coated with cellular metabolites, and extruded by the cells into the incubation media [6]. Targeted metabolomic analyses of compounds isolated from *Arabidopsis thaliana* plants using this method, which we named nanoharvesting, showed enrichment of specific flavonoids, compounds belonging to a large subgroup of polyphenolic plant natural products called phenylpropanoids [6,7]. We showed that flavonoids with a catechol ring were more abundant in NP bio-coronas than flavonoids without the catechol ring [6]. Untargeted metabolomic analyses of the bio-coronas formed on the surface of TiO_2_ NPs incubated with Arabidopsis tissues confirmed that flavonoids are preferred ligands but also showed that lipids and, in particular, fatty acids are avidly bound to the NP surface, thus competing with flavonoids [8].

Here, we applied the nanoharvesting/untargeted metabolomic analyses pipeline to determine the identity of the metabolites that are enriched in the bio-coronas formed after co-incubation of ultra-small anatase TiO_2_ NPs with tissues of two non-model plant species, *Ocimum sanctum* L. (Tulsi; holy basil) and *Rubia tinctorum* (common madder). Tulsi is a medicinal plant traditionally used in India and nowadays, also in complementary alternative medicine approaches [9]. Tulsi, described as “a herb for all reasons” [10], is considered to be a potent adaptogen, a herb that helps with the adaptation to stress and promotes homeostasis [9–19]. It was only recently that Tulsi was subjected to comprehensive molecular analyses such as metabolomics and transcriptomics [20–23]. The common or dyer's madder is a perennial plant species belonging to the coffee family and is best known as a source of an anthraquinone-type red dye, which is extracted from roots [24]. Many anthraquinones synthesized in plants have therapeutical value as they have antimicrobial, anti-inflammatory, and anti-oxidant properties [25]. Anthraquinones-rich madder root extracts are used globally in traditional treatments of several conditions, most notably, for treatments of kidney stones and urinary tract disorders [24].

We found that the bio-coronas of anatase TiO_2_ NP incubated with Tulsi leaves and madder roots are rich in flavonoids, lipids, and peptides. Together with the conclusions of our prior study [8], we show that nanoharvesting using anatase TiO_2_ NP from any plant source can be used to enrich flavonoid and lipid compounds, which can then be used in targeted bio-assays.

## Main text

### Materials and methods

#### Plant growth

Tulsi (*Ocimum sanctum* L. Rama also known as *O. tenuiflorum* L. Rama [26]) and common madder (*Rubia tinctorum* L.) seeds were obtained from https://strictlymedicinalseeds.com/, and plants were grown in the field. Young leaves of non-flowering Tulsi plants were harvested and used for the analyses. Roots of second-year madder plants were used for the analyses.

#### Extraction procedure

A pool of three young leaves excised from separate plants or a mix of root segments from different plants (~ 100 mg tissue per sample) was used for both the methanolic extraction and nanoharvesting using a previously described method [8]. In brief, to obtain methanolic extracts, tissues were frozen in liquid nitrogen, disrupted using zirconium beads and a bead beater in 10 volumes of 1% HCl/methanol, and incubated in acid methanol for 16 h in the dark at 4 °C. Samples were then centrifuged, subjected to chloroform partitioning, and the methanolic phase was used for the analyses. Nanoharvesting was done using an aqueous dispersion of anatase TiO_2_ NPs obtained from US Research Nanomaterials Inc. (15% wt, 1.9 M). This TiO_2_ NPs stock solution was diluted in LC–MS-grade water and sonicated in the sonification water bath for 2 min immediately before nanoharvesting. The size distribution, composition, hydrodynamic diameter, and Zeta potential of the NPs were previously described [27]. For nanoharvesting, tissue was immersed in 1 ml of 1.9 mM TiO_2_ NPs suspension and co-incubated on a platform rocker (10 rpm) for 4 h at 22 ºC in the dark. The tissues were then removed, and the coated NPs were pelleted (1 min, 3500 rpm, 22 ºC). For elution of compounds bound to the particle surface, 100 µl of 1% HCl/methanol was added to each pellet. Pellets were disrupted by 1 mm zirconium beads in a bead beater (2 min at 4000 rpm) and sonicated for 2 min. Samples were then mixed with an equal volume of chloroform, vortexed, and centrifuged (2 min at 4000 rpm). The upper methanolic phase was used for the analyses. Before the LC–MS/MS analyses, all samples were filtered through 0.22 micron filters (Cameo 3 N, GE Waters).

#### Untargeted metabolomics analyses

Untargeted MS analysis was performed at the Proteomics & Mass Spectrometry Facility at the Danforth Plant Science Center, as previously described [8]. For data processing, datasets were analyzed using MetaboAnalyst 3.0 (www.metaboanalyst.ca [28]) and the R package *ComplexHeatmap* [29] using previously described parameters [8].

### Results and discussion

For both plant species, after the identification of isolated metabolites, we compared the composition of the methanolic extract with the composition of the bio-coronas to determine whether specific chemical classes of metabolites are more enriched by one of the methods. The metabolite identification was done using the Elements software package (http://www.proteomesoftware.com), and the metabolites with a minimal identity (ID) score of 0.5 were used in further analyses.

**Fig. 1:**
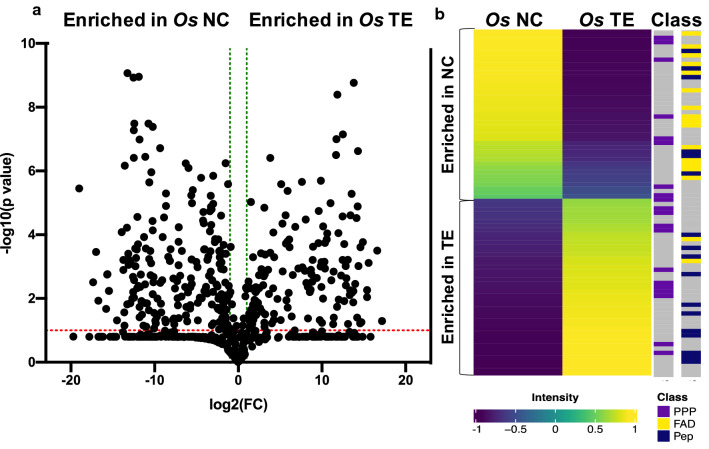
Comparative analyses of the composition of the bio-corona (nanoconjugates, NC) and the total methanolic extract (TE) of *Ocimum sanctum* (Tulsi; *Os*) leaves. **a** Volcano plot of metabolites with an identity (ID) score of ≥ 0.5. Fold change (FC) of 2 is marked with a green dotted line and *p-*value of 0.1 is marked with a red dotted line. **b** The heatmap was constructed from the average normalized intensity values of significant metabolites identified in (**a**) with FC > 2, *p* > 0.1, and an ID score ≥ 0.9. Hierarchical clustering analysis was done using the Manhattan clustering distance and ward.D2 clustering method. The color intensity scale is positioned below the heatmap. Different chemical classes are labeled with different colors in the legend positioned on the left-hand side. Fatty acid derivatives (FAD) are marked in yellow on the right-hand sidebar, phenylpropanoids (PPP) in purple, peptides (Pep) in blue and compounds that belong to other chemical classes in gray

For Tulsi, a set of 617 endogenous metabolites was used as an input for MetaboAnalyst. We first did univariate analysis with the volcano plot method, which combines Fold Change (FC) analysis and *t*-test, to identify metabolites that are significantly more abundant in methanolic extracts and metabolites that are significantly more abundant in bio-coronas (Fig. 1a). This approach identified a subset of 341 metabolites that significantly accumulated (FC ≥ 2 and P ≤ 0.1) using either extraction method. These significant metabolites were sorted into chemical classes following the Human Metabolome Database (HMDB, http://www.hmdb.ca/) classification. The largest fraction of metabolites that were significantly differently extracted by methanol and nanoparticles from Tulsi leaves were fatty acids and their derivates (34.29%), followed by flavonoids and other phenylpropanoids (19.05%), and finally, peptides (18.10%). Next, we performed hierarchical clustering analysis on those metabolites with an ID score ≥ 0.9 (n = 88). These analyses showed that whereases some flavonoids were more abundant in nanoharvested extracts, others were nanoharvested but with a lower efficiency since they were more abundant in methanolic extracts (Fig. 1a). The most abundant nanoharvested flavonoid was identified as 5,3′-dihydroxy-6,7,4′-trimethoxyflavone (ID score = 1; mass accuracy score = 0.99; isotope distribution score = 0.99), a species that does not have a catechol ring but has six oxygen atoms in the vicinal position that are likely to be responsible for the binding to the NP surface. The fatty acid that was most efficiently nanoharvested was identified as 9,10,-dihydroxy-12*Z*-octadecanoic acid (9,10-DiHOME; ID score = 0.977; mass accuracy score = 0.98; isotope distribution score = 0.98). Isolated peptides were either dipeptides (33.3%) or tripeptides (66.7%), and they were enriched in basic amino acids at the C-terminal end, with 50% of peptides having arginine and 17% having lysine as the C-terminal amino acid residue (Additional file 1). Significant binding of arginine through electrostatic interaction and hydrogen bonds of the arginine guanidinium protons to the TiO_2_ NP surface oxygen atoms was previously described in vitro and is believed to be essential for the attachment of sensitizing proteins (e.g., bacteriorhodopsins) to TiO_2_ solar cells [30]. Here we show that arginine- and lysine-containing peptides preferentially bind to the surface of TiO_2_ NP even when they are a part of a complex mixture of metabolites.

**Fig. 2:**
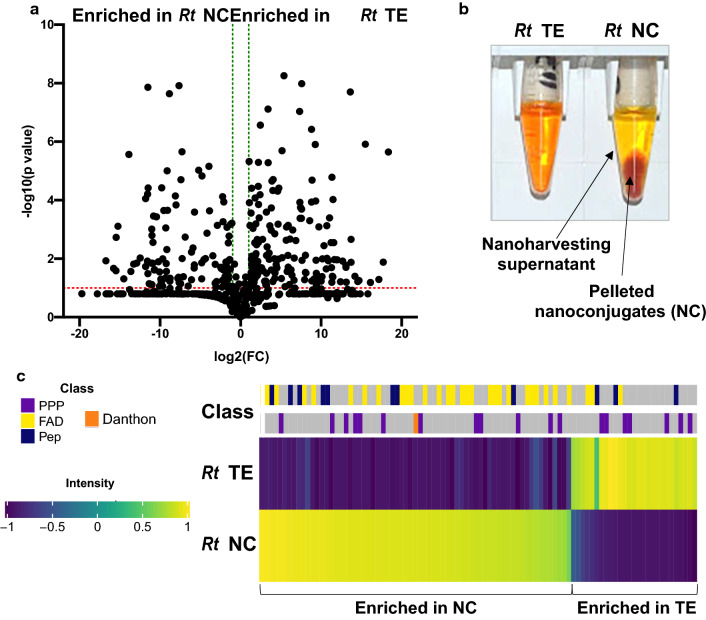
Comparative analyses of the composition of the bio-corona (nanoconjugates, NC) and the total methanolic extract (TE) of common madder (*Rubia tinctorum* L.; *Rt*) roots. **a** Volcano plot of metabolites with an ID score ≥ 0.5. Fold change (FC) of 2 is marked with a green dotted line and a *p-*value of 0.1 is marked with a red dotted line. **b** Visual characteristics of methanolic extract (TE) and the nanoharvested solution, which was briefly centrifuged to pellet the nanoconjugates (NC), which were then used for the acid/methanol-based stripping of the adsorbed metabolites. **c** The heatmap was constructed from the average normalized intensity values of metabolites shown to be significantly different using the volcano plot method. The color intensity scale is on the left-hand side of the heatmap. Different chemical classes are labeled: fatty acid derivatives (FAD) in yellow, phenylpropanoids (PPP) in purple, peptides (Pep) in blue, anthraquinone danthon in orange, and compounds that belong to other chemical classes in gray

Analyses of the common madder extracts followed the same steps as described for Tulsi. A set of 610 endogenous metabolites (ID ≥ 0.5) was used as an input for MetaboAnalyst, and after analyses using the volcano plot method (Fig. 2a) and sorting of the significant metabolites (n = 176) using the HMDB, we determined that the chemical profile of the metabolites that were differently extracted by methanol and NPs from madder roots was strikingly similar to those in Tulsi: 32% were fatty acids and their derivates, followed by flavonoids and other phenylpropanoids (21%), and finally, peptides (12%). Isolated peptides were either dipeptides (23%) or tripeptides (77%), and 84% of peptides had either lysine or arginine as a C-terminal amino acid. Since the number of significant metabolites with ID ≥ 0.9 was relatively low (n = 19), we performed the hierarchical clustering analysis on the whole subset of significant metabolites regardless of the ID score. The most abundant nanoharvested flavonoid was identified as 4,2′-dihydroxy-3-methoxy-5′-methylchalcone (ID score = 0.57; mass accuracy score = 0.78; isotope distribution score = 0.42). The fatty acid that was most efficiently nanoharvested was identified as ricinoleic acid (ID score = 0.97; mass accuracy score = 1; isotope distribution score = 0.99). All peptides enriched in nanoharvested extracts had either lysine (37.5%) or arginine (62.5%) as a C-terminal amino acid (Additional file 2), confirming that anatase TiO_2_ NP-based nanoharvesting can be used for the enrichment of peptides that have C-terminal lysine or arginine from any plant extract. Despite the intense color of both the methanolic extract and the nanoharvested pellet (Fig. 2b), none of the yellow/red dye compounds known to be present in common madder roots have been identified with an ID score ≥ 0.5. The only identified anthraquinone was 1,8-dihydroxyanthraquinone (danthon; ID score = 0.76; mass accuracy score = 1; isotope distribution score = 0.96), a red anthraquinone derivative, and this compound was enriched in nanoconjugate extracts (Fig. 2c). More than sixty different colored anthraquinones, such as Alizarin and purpurin, and anthraquinone glucosides have been identified in *Rubia* species [31]. However, it has been recently questioned how many of these compounds are present *in planta* and how many are tissue storage, extraction, or isolation method artifacts [32]. Considering the color of the nanoconjugate pellet, sensitivity of the method we used, and the fact that Alizarin and its derivatives are known to efficiently bind to the surface of TiO_2_ NPs [33–35], it was surprising that we did not identify any of the known madder dyes in TiO_2_ NP bio-coronas. This finding strengthens the claims that the extraction method, sample processing time, and possibly the sample complexity can lead to the misidentification of madder dyes.

## Limitations

Plants have always been a rich source of affordable therapeutic compounds and lead compounds for developing new medications. Coupling nanoharvesting, a fast and selective method of isolation of metabolites, with untargeted metabolomic profiling promises to make the isolation and identification of biologically active compounds more efficient. Nanoparticle-based “fractionation” of the sample, followed by—on the one hand—activity assays and on the other hand, metabolomic profiling may facilitate the identification of active compounds, particularly if they belong to a specific chemical class (flavonoids, lipids, and peptides in case of anatase TiO_2_ NP-based nanoharvesting).

The limitation of this coupled methodology is, however, a combination of the limitations of both methods. Upon entry into a cell, NPs are coated with molecules that have a high affinity for the NP surface and they form a tightly bound monolayer called hard bio-corona [36,37]. That implies that a compound of interest may not be efficiently nanoharvested if another metabolite with equal or higher affinity for the NP surface is present in the sample at a higher concentration as the higher-affinity metabolite will become a major component of the hard bio-corona and saturate the NP surface, Limitations of untargeted metabolomic approaches have been discussed in detail (e.g., [38]), and they include sample preparation artifacts and misidentification of complex metabolites (e.g., plant secondary metabolites). Irrespective of the limitations, the bioassay-guided, NP-based fractionation of extracts of medicinal plants coupled with untargeted metabolomic analyses is a promising approach for future identification of bioactive compounds.

## Supplementary Information

**Additional file 1: Table S1.** List of significant metabolites enriched in bio-coronas of NPs isolated from Tulsi leaves. Names of the compounds, their chemical class according to the Human Metabolome Database (HMDB, http://www.hmdb.ca/) classification, and chemical class coding used for hierarchical clustering analysis shown in Fig. 1b are shown.

**Additional file 2: Table S2.** List of significant metabolites enriched in bio-coronas of NPs isolated from madder roots. Names of the compounds, their chemical class according to the Human Metabolome Database (HMDB, http://www.hmdb.ca/) classification, and chemical class coding used for hierarchical clustering analysis shown in Fig. 2c are shown.

## Data Availability

All research materials are commercially available and all data is available upon request.

## Abbreviations

NPs: Nanoparticles
TiO_2_: Titanium dioxide
